# Intelligent Prediction and Numerical Simulation of Landslide Prediction in Open-Pit Mines Based on Multi-Source Data Fusion and Machine Learning

**DOI:** 10.3390/s25103131

**Published:** 2025-05-15

**Authors:** Li Qing, Linfeng Xu, Juehao Huang, Xiaodong Fu, Jian Chen

**Affiliations:** 1 Faculty of Land Resources Engineering, Kunming University of Science and Technology, Kunming 650093, China; 18756526353@163.com; 2 State Key Laboratory of Geomechanics and Geotechnical Engineering Safety, Institute of Rock and Soil Mechanics, Chinese Academy of Sciences, Wuhan 430071, China; jhhuang@whrsm.ac.cn (J.H.); xdfu@whrsm.ac.cn (X.F.); chenjian@whrsm.ac.cn (J.C.); 3 University of Chinese Academy of Sciences, Beijing 100049, China

**Keywords:** high and steep slopes, terrain-following flight, multi-source data fusion, machine learning, numerical simulation, landslide prediction

## Abstract

With the increasing mining depth, the stability of open-pit mine slopes has become an increasingly important concern. This study focuses on an open-pit mine in Southwest China and utilizes unmanned aerial vehicle (UAV) technology to gather data from these high and steep slopes. First, high-precision digital surface models and digital orthophoto data are collected using UAV terrain-following flight technology. However, two major challenges arise when applying geographic information systems (GISs) to this issue. The first challenge is that the extreme steepness of the slopes causes overlapping lithological layers at the same location, which GISs cannot resolve. The second challenge is that GISs cannot assess the influence of faults on landslides by calculating three-dimensional spatial distances. To overcome these issues, this study proposes the construction of a detailed 3D geological model for the entire mining area. This model allows for a more precise analysis of the lithology and fault spatial distances. A GIS is then applied to analyze the slope, curvature, and slope direction. Multi-source data fusion is employed to link spatial coordinates and create a dataset for further analysis. Five machine learning models for landslide prediction are compared using this dataset. Based on these comparisons, a high-precision random forest and slope boosting coupled method is developed to enhance the landslide prediction accuracy. Finally, a numerical simulation of a regional focus area is conducted, simulating the excavation process of an open-pit mine and analyzing the timing, location, and state of potential landslides. The results indicate that combining machine learning and multi-source data fusion provides a highly accurate, efficient, and straightforward method for landslide prediction in the high and steep slopes of open-pit mines.

## 1. Introduction

As mining depths increase, many open-pit mines are adopting deep concave mining methods, which significantly increase the slope height and slope, thereby reducing the overall slope stability. Geological surveys on these steep slopes are challenging due to the complex geological conditions and the high risk of hazards. Moreover, the constantly changing topography in open-pit mines complicates the timely and accurate measurement and updating of slope geological data. Traditional measurement techniques and analysis methods can no longer provide the required accuracy, efficiency, or predictive capabilities.

Recent advancements in UAV measurement technology offer a promising solution for the acquisition of slope models and parameters. Xi W [1] used UAVs to capture images of steep slopes, applying the scale-invariant feature transform (SIFT) algorithm to extract feature points and the RANSAC algorithm for matching. This was followed by stereoscopic image stitching to create an orthophoto map and a digital terrain model (DEM). Comparing these UAV-generated DEMs with total station measurements revealed minimal elevation errors, confirming their accuracy advantage. Albarelli [2] utilized UAV-acquired high-resolution 3D point cloud data to extract a rock quality index (RQI) for local-scale rockfall susceptibility assessment. Hill [3] combined photogrammetry with light detection and ranging (LiDAR) technology and integrated it into GIS platforms. Q Li [4] used UAV imagery combined with computer vision techniques for slope monitoring, while H Huang [5] analyzed changes in digital surface models (DSM) to detect and quantify slope deformation. These studies demonstrate that UAV-based remote sensing methods are fast, safe, effective, and particularly well suited for the monitoring of steep, cloudy environments.

For steep slopes in large-scale deep concave open-pit mines, the rapid integration and efficient analysis of multi-source data remain a challenging task. Jie Yang [6] proposed a data fusion method based on time-varying monitoring and high-precision numerical simulation, providing new ideas for dam safety and stability analysis. Several researchers [7,8] have used mathematical or statistical methods to compare field-measured displacements with simulation results to assess slope stability. These models include nonlinear models [9,10] and statistical models [11,12]. However, most current landslide prediction and early warning models primarily analyze deformation data from a single measurement point. While this approach reflects the local stability, it fails to provide a comprehensive analysis of the interrelationships between multiple measurement points and the overall slope condition [13].

As GIS technology continues to develop, GIS-based landslide susceptibility mapping has become widely used in landslide analysis. Wang X [14] employed models such as logistic regression, hierarchical analysis, and frequency ratios to select factors like the slope, slope direction, land cover, precipitation, and lithology and their spatial relationships with roads, drainage systems, and faults for a comprehensive analysis. These datasets were integrated into a GIS platform for visual modeling. Ji Jian et al. [15] developed a GIS-based automatic analysis tool for regional landslide susceptibility using Python, successfully applying it in engineering projects. Zhang Jikai et al. [16] conducted a comparative study of disaster-causing factors and evaluation models in landslide disasters in Jiuzhaigou, concluding that coupled models generally have better accuracy than individual models.

Simultaneously, machine learning and deep learning models have shown great promise in landslide prediction. Random forest (RF) [17], logistic regression (LR) [18], support vector machine (SVM) [19], convolutional neural networks (CNN) [20], and other models are effective in capturing complex nonlinear interactions between triggering factors and landslide occurrences [21]. Huang, Invention et al. [22] constructed two sample datasets based on the spatiotemporal characteristics of landslide prediction events and used them to train and evaluate several machine learning models, such as SVM, a multi-layer perceptron (MLP), and RF, providing a reliable framework for prediction in various geological environments. Wang W. [23] established a landslide sensitivity index system for the study area and compared multiple machine learning algorithms. The results showed that the RF model had the highest prediction accuracy, with an overall correct rate of 98.00%.

Bao et al. [24] proposed a three-dimensional numerical model based on the coupling of SPH and FDEM to simulate the interaction between debris flows and structures, enabling a quantitative analysis of structural damage. Troncone et al. [25] compared the applications of the coupled Eulerian–Lagrangian method, material point method, and SPH in analyzing the post-failure stages of flow-type landslides, highlighting their effectiveness in addressing large deformation problems. Pastor et al. [26] introduced an enhanced two-phase debris flow propagation model that incorporated SPH nodes and a depth-integrated approach, considering the impact of the pore water pressure on the debris flow velocity, providing more accurate predictions.

Building upon previous research, this study proposes a novel approach to predicting landslides on slopes in open-pit mines. The method offers several key advantages: (1) UAV-based terrain-following flight technology is employed to acquire high-precision surface data, which avoids the risks of traditional methods and improves the accuracy of standard UAV imaging; (2) a GIS is applied to open-pit mining, taking full advantage of its speed, high accuracy, and powerful update capabilities; (3) a refined 3D geological model of the mining area is established through multi-software coupling; (4) machine learning methods are introduced to model and predict landslides based on the fused dataset, enabling a comprehensive assessment of slope hazards and generating interpretable visual outputs; (5) numerical simulations are conducted for key areas to analyze the specific location, timing, and intensity of potential landslides, thereby ensuring the safety of mining operations.

## 2. Problems in the Selection and Application of Landslide Factors

### 2.1. Selecting Landslide Evaluation Factors Based on GIS

**Figure 1:**
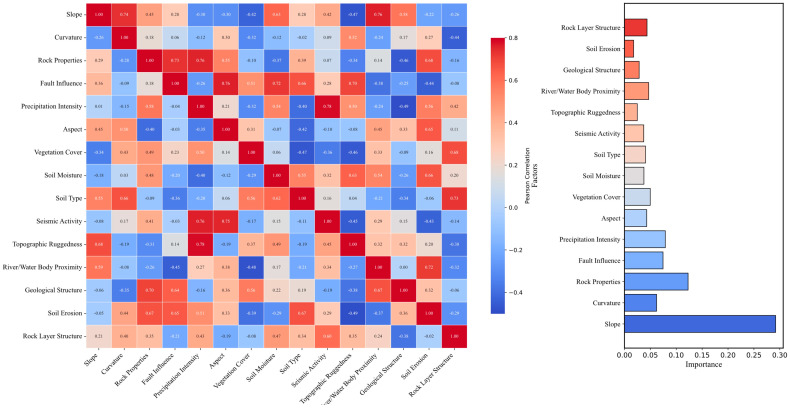
Correlation and importance map of evaluation factors.

The accuracy of landslide prediction primarily depends on the selection of evaluation factors, and choosing these factors wisely is crucial. Numerous previous studies have explored the importance [27,28,29,30,31] and relevance [32,33,34,35,36,37,38,39,40,41] of various factors related to landslides, producing relatively consistent results. Some researchers have used principal component analysis (PCA) to assess the significance of multiple factors and have assigned specific values to them. This is often combined with field geological surveys, histograms depicting the importance of related factors, and corresponding heat maps, as shown in Figure 1.

In this study, five influencing factors—the slope, curvature, lithology, fault influence, and slope direction—are selected for further analysis. To ensure objectivity and accuracy, the study focuses specifically on these five factors. GIS technology can be applied to assess the slope, curvature, and slope direction. Using the ArcGIS 10.8 software, these factors can be extracted from the high-precision digital elevation model (DEM) generated by UAVs. Based on the grid size division formula proposed in the literature, the following formula can be applied:

(1) $$G_{s}=7.49+0.0006S^{2}\times 10^{-9}S^{2}+2.9\times 10^{-15}S^{3}$$

In the formula, *G_s_* represents the appropriate grid size, and *S* represents the data scale. In this study, a grid size of 5 m × 5 m is used for landslide susceptibility assessment, with a total of 89,531 grid cells. A 5 m resolution grid effectively captures the probability characteristics of landslides, while avoiding the slower processing speeds and higher hardware demands that may arise from using smaller grid sizes.

### 2.2. Problems and Solutions in Applying GIS for Landslide Prediction in Open-Pit Mines

GIS technology has proven to be an effective tool for landslide prediction due to its speed, cost-effectiveness, and ability to stay up-to-date. It is particularly suitable for large open-pit mining areas. However, in traditional GIS analysis, two key issues remain unresolved. The first is the challenge presented by high and steep slopes, where multiple lithologies coincide at the same location—a problem that GISs cannot handle. The second issue is that GISs are unable to calculate the three-dimensional spatial distances needed to assess the effects of faults on landslides. The influence of faults on landslides has not been thoroughly examined, and this limitation becomes especially apparent when GIS technology is applied to open-pit mining analysis.

**Figure 2:**
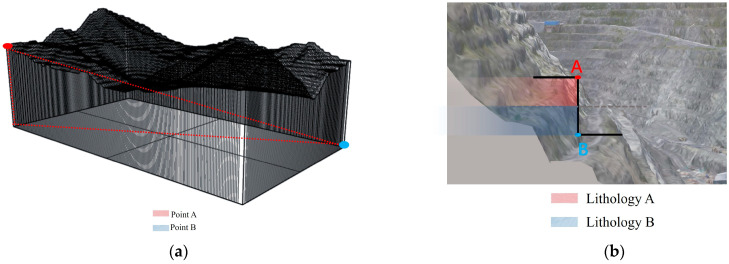
Three-dimensional data fusion technical challenges: (**a**) spatial distance; (**b**) misidentification of lithology.

As shown in Figure 2a, traditional GIS analysis in the complex spatial structure of an open-pit mining area typically relies on planar distances. However, for deeper excavations, spatial distances can differ significantly from planar distances, leading to potential biases or substantial errors in the analysis. Additionally, in mining areas with steep or near-vertical slopes, traditional 2D GIS techniques face limitations in lithology identification. As shown in Figure 2b, in such cases, GISs may only recognize lithology A, when, in reality, both lithology A and lithology B coexist in the area. These two lithologies may exhibit distinct landslide mechanisms. Relying solely on traditional GIS methods could overlook these crucial lithological differences, ultimately affecting the accuracy of landslide risk assessments.

To overcome the limitations of traditional methods in combining 3D geologic modeling with spatial data analysis, this study proposes an innovative approach that integrates 3D geologic modeling, as shown in Section 4, with GIS data processing functions, as shown in Section 5. Specifically, the GIS data interface is used to extract the spatial coordinate information from raster data, obtaining the coordinates of the center point, where the Z value represents the elevation. Additionally, the coordinates of a specific vertex can be obtained, allowing for the precise location of a point within the 3D geologic model. Data fusion is then carried out by matching these spatial coordinates.

## 3. Terrain-Following Flight-Based Data Acquisition Using Unmanned Aerial Vehicles

### 3.1. Principle of Terrain-Following Flight Technology

A large open-pit mine in Southwest China, with slopes of up to 600 m, faces measurement challenges due to complex terrain. Traditional methods are inefficient and pose safety risks. Terrain-following flight (TFF) technology, maintaining a constant altitude relative to the ground, effectively addresses these challenges in areas with variable elevation.

**Figure 3:**
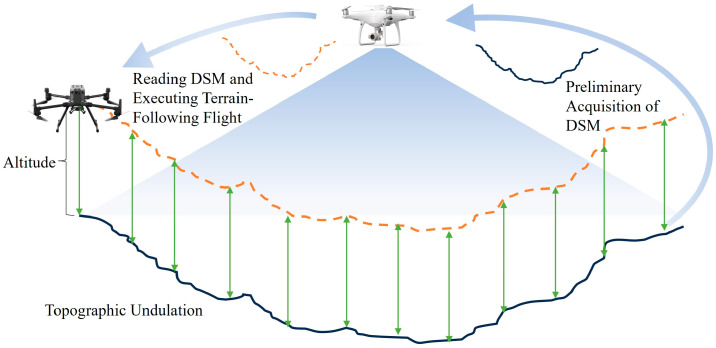
Schematic diagram of UAV terrain-following flight.

In aerial surveying, the operational procedures for TFF are especially crucial. As shown in Figure 3, the UAV maintains a constant altitude relative to the ground surface by assigning a fixed vertical offset based on preloaded 3D terrain data (DSM). The UAV autonomously adjusts this altitude throughout the flight. This method significantly simplifies the complex process of designing layered flight paths in traditional surveys, greatly improving the measurement accuracy while ensuring that the UAV maintains a safe operating distance from mine machinery. Compared to standard 2D aerial photogrammetry, TFF can achieve millimeter-level mapping accuracy. The UAV’s dynamic ability to adapt to terrain changes, coupled with improved positioning accuracy, ensures continuous and complete data collection, minimizing errors caused by data gaps and elevation fluctuations.

### 3.2. Data Collection Processy

**Figure 4:**
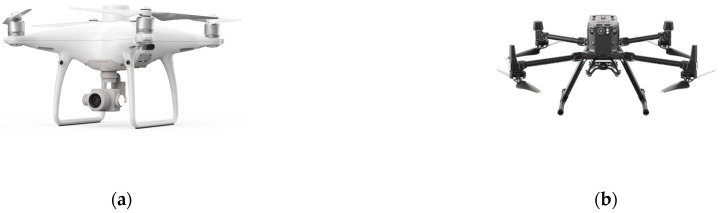
UAV equipment for terrain-following flight: (**a**) DJI PHANTOM 4 RTK UAV (DJI, Shenzhen, China); (**b**) DJI Matrice 300 RTK UAV (DJI, Shenzhen, China).

The topographic data acquisition for the open-pit mine began with a field survey to gather detailed information. Seventeen image control points, including three for validation, were deployed across the mine using the WGS84 coordinate system. Two-dimensional aerial surveys were conducted with a DJI Phantom 4 RTK drone, As shown in Figure 4a, and images were processed with the M3D v10.x software to generate DSMs in TIFF format. These datasets were then imported into the DJI GSR APP for TFF operations, ensuring accurate data collection across the mining area.

During the flight planning and execution phases, a detailed TFF route was developed based on the processed DSM data. The UAV maintained a fixed altitude of 60 m above the ground throughout the mission, ensuring consistent and accurate data collection. The flight routes were generated using DJI Pilot, which automatically created TFF paths by integrating terrain data and user-defined flight parameters to optimize the flight trajectory and ensure comprehensive spatial coverage. For equipment, a DJI Matrice 300 RTK drone was used, As shown in Figure 4b. Prior to takeoff, the RTK system was thoroughly checked to ensure stable signal reception and positioning accuracy, ensuring the safety and reliability of the flight operation.

### 3.3. UAV Data Modeling and Output Results

During the office processing stage, 10,130 field images were imported into ContextCapture and processed using the “structure from motion” method. Feature points were extracted and matched between images to generate a sparse point cloud through multi-view geometry. A high-density point cloud was created using multi-view stereo, and the photogrammetric accuracy report confirmed that errors were within 1 pixel, ensuring the quality of the process.

The Poisson surface reconstruction method converted the point cloud into a polygonal mesh, onto which image textures were mapped to generate a 3D model. The model’s accuracy was verified using GPS, resulting in the creation of a DSM and DOM. DSMs show surface feature heights, while DOMs provide detailed, accurate image features.

**Figure 5:**
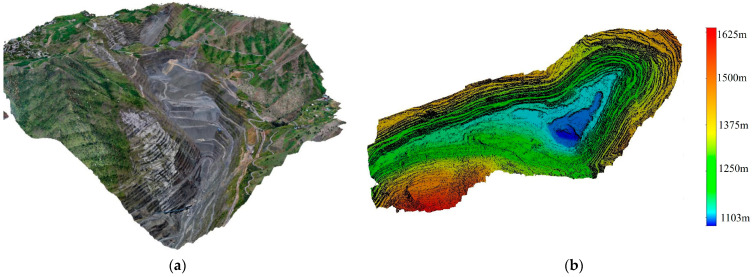
Preliminary results of UAV-based modeling: (**a**) DOM of the entire mining area; (**b**) DSM of the open-pit mining area.

This study output models including contour data, 3D point clouds, DOMs, and DSMs (Figure 5). Each model was exported in a suitable format for its intended use: DOMs in OSGB format, 3D point clouds in LAS format, and contour data in STL format. These standardized outputs ensure compatibility with engineering, GIS, and 3D modeling platforms, enabling seamless integration into further workflows.

## 4. Refined 3D Geological Modeling Based on Multi-Source Data Fusion

### 4.1. Preliminary Geological Model Construction Method Based on 3DMine

**Figure 6:**
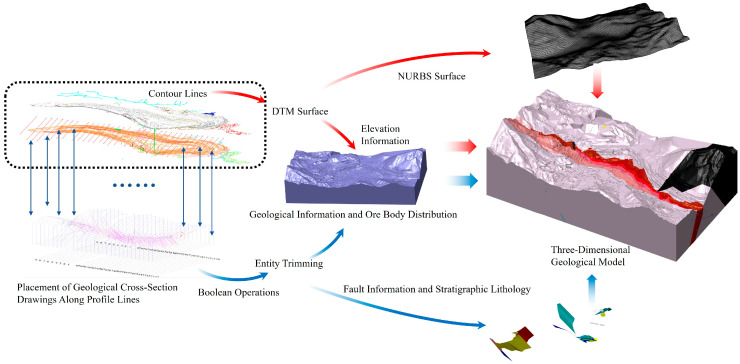
The detailed flowchart for refined 3D geological modeling.

The contours generated by the UAV were imported into 3DMine and used as constraints to construct a digital terrain model (DTM) of the mine site through Boolean operations. The 3DMine tool can convert mining data into intuitive 3D graphical models, and the software supports the import and export of various types of data. As shown by the blue arrows at the bottom of Figure 6, the DTM serves as a cutting surface to trim the predefined rectangular volume, resulting in an accurate and intuitive surface representation of the mine site. The quality of the DTM directly affects the accuracy of the final 3D geological model.

To achieve geological stratification and lithological attribution, stratigraphic interfaces were constructed based on 39 geological cross-sections (P1–P9, P70–P89, NES0–NE3, ES1–ES6) extracted from the current mining plan. Using 3DMine’s spatial positioning, the profiles were aligned with the surface model, and internal stratigraphic data were imported to trim the external model for consistency with geological information.

After importing and simplifying the profiles, the “triangle mesh within closed polylines” function was applied to create fault boundaries. Fault modeling used detailed fault data, with 3DMine’s open polyline triangulation feature enabling precise fault location modeling. Local pruning and computational timing techniques minimized errors and ensured consistency in geological feature representation. After validation, the preliminary 3D geological model was completed, providing a foundation for further mining analysis and decision-making.

### 4.2. Refinement and Correction Method for Geological Models Based on Rhino

The models exported from 3DMine were optimized in Rhino to address issues such as excessive DTM surface areas and detail redundancy. Rhino supports highly precise curve, surface, and polygonal modeling and can handle complex 3D geometries, making it suitable for designing intricate shapes. Therefore, we chose the Rhino 8 software for detailed processing. By merging coplanar meshes, both the modeling and computational efficiency were significantly improved. Adjusting the quadrilateral reconstruction parameters resulted in a more streamlined surface model of the mining area, as shown by the red arrows in the upper part of Figure 6. Areas with broken or overlapping meshes were manually removed, added, and joined, and these areas were checked and repaired in real time using rendering and shading modes.

The final step involved creating a continuous surface model using Rhino’s NURBS (Non-Uniform Rational B-Spline Curve) modeling feature, providing a smooth and accurate representation of the mining area for further analysis and simulation. By integrating drill hole data, cross-section profiles, and contours from various sources, a comprehensive 3D geological model with lithological and fault characteristics was developed. The conversion from triangular meshes to NURBS surfaces produced a model compatible with visualization and analysis tools, forming the foundation for subsequent geological modeling and engineering applications, enabling detailed analysis, resource assessment, and mining operation planning.

## 5. Processing of Surface Information in Mining Areas Using GIS Technology

### 5.1. Landslide Point Cataloging Based on GIS

Historical landslide data, geological surveys, and previous scientific analyses have provided important information for the identification and delineation of the main landslide-prone areas, lithological conditions, and fault distributions within the mining area. However, these historical datasets have limitations when it comes to detecting and recording small-scale landslide events. To address this, two years after the initial survey, a second UAV aerial survey was conducted using the same methods as in the first. We collected updated topographic data and used the ArcGIS software to convert the DSM data from both time points into DEMs for subsequent comparative analysis.

**Figure 7:**
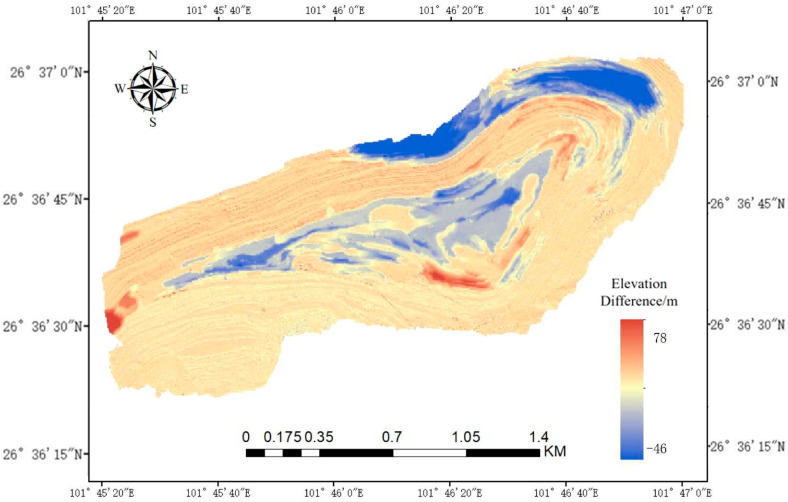
DEM of differences between two time periods.

The DEM data from the two different time points across the entire mining area were compared using a raster calculator, resulting in a height difference map, as shown in Figure 7. These height differences visually reflect the occurrence of landslides, with the map accurately displaying the elevation changes between the two time points. This helps to identify landslide events and their affected areas. Through comparative analysis, the dynamic characteristics of landslide activity can be revealed, providing a basis for future landslide management and prevention. By combining historical landslide data with field observations, landslide events and their specific locations can be more accurately recorded. Ultimately, through this comprehensive analysis, a total of 4064 landslide points were identified.

### 5.2. Landslide Factor Analysis Based on GIS

Geographic information system (GIS) technology can be used to process slope, curvature, and slope direction factors. Specifically, the high-resolution DEM generated by UAV surveying can be imported into the ArcGIS software to extract these topographic parameters. Using spatial analysis tools, the “Slope”, “Curvature”, and “Aspect” modules in ArcGIS can accurately derive these factors, providing important input data for landslide susceptibility assessment.

**Figure 8:**
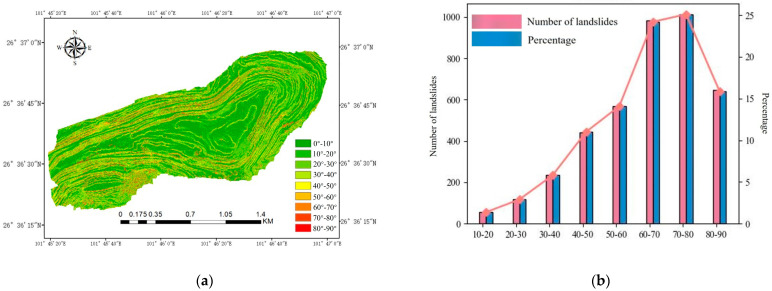
Slope and landslide points: (**a**) slope distribution map of the open–pit mining area; (**b**) number and proportion of landslides of different slopes.

Slope is a key factor in landslide occurrence, playing a decisive role in determining the probability of landslides. Variations in slope directly affect the stability of the slope, significantly influencing the likelihood and intensity of landslide events. In steep slope conditions, the slope stability decreases, which may increase both the probability and severity of landslides. Historical disaster points and height difference maps indicate that landslide disasters primarily occur in high-slope areas. Figure 8 illustrates the statistical relationship between slopes with different slope values and the distribution of landslide points, as well as a visual representation of these data.

**Figure 9:**
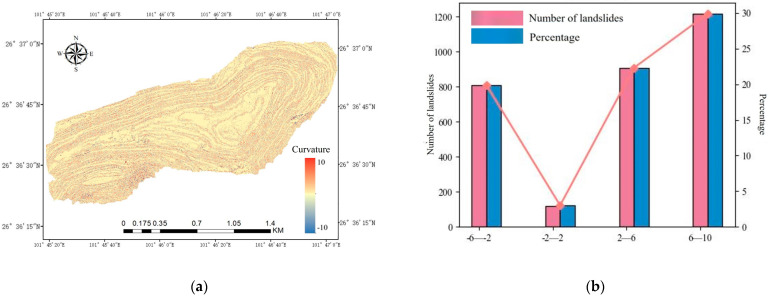
Curvature and slip points: (**a**) curvature distribution map of the open-pit mining area; (**b**) number and proportion of landslides of different slopes.

The slope curvature reflects the topographic features of an area and is an important indicator in measuring the degree of surface curvature, which is highly relevant to landslide studies. The profile curvature affects the water flow speed, soil moisture, and erosion intensity, all of which influence the landslide risk. When the profile curvature is negative, the slope is concave, making it more likely to collect water, which increases the soil moisture and landslide risk. Conversely, when the profile curvature is positive, the slope is convex, leading to water runoff, drier soil, and a lower risk of landslides. Areas with higher absolute values of profile curvature are more prone to landslides. Figure 9 illustrates this relationship.

**Figure 10:**
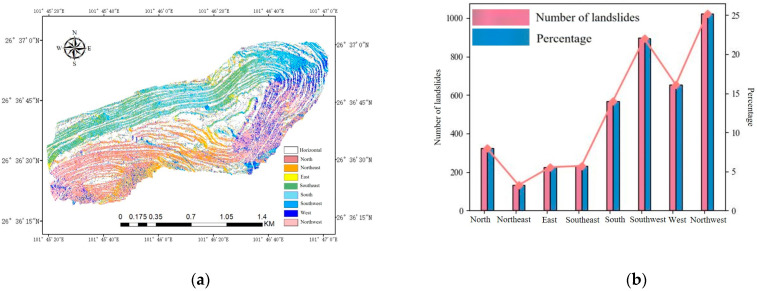
Aspect and slip points: (**a**) aspect distribution map of the open-pit mining area; (**b**) number and proportion of landslides of different slopes.

The slope orientation is also a significant factor in open-pit mining. In large, deep open-pit mines, many of the haul roads and working faces are located at the base of the slope. These areas can be considered flat, with little to no dip. Data indicate that the probability of landslides in these flat areas is nearly zero. On the other hand, historical landslide data show that landslides occur more frequently and with greater intensity in specific areas, particularly to the southeast, east, and south. Figure 10 illustrates this pattern.

### 5.3. Multi-Source Data Fusion

This paper integrates the 3D geological modeling described in Section 3 with the GIS data processing functions presented in Section 4. Specifically, the GIS data interface is used to extract spatial coordinate information from raster data, allowing the DSM coordinates of the center point to be obtained, where the Z value represents the DSM elevation. The point information, which includes 3D spatial coordinates, has a unique corresponding point in the 3D geologic model. The data are then fused using the same method as for the 3D coordinates.

During the coordinate conversion process, a spatial reference system conversion tool is employed to ensure accurate mapping between the model coordinate system in Rhino and the WGS84 geographic coordinate system used in ArcGIS. This ensures spatial consistency throughout the data transmission process. In the Rhino modeling environment, scripting tools are used in combination with Rhino’s development interface to process and transform the extracted data, accurately converting the geographic coordinate data into 3D point objects necessary for modeling. Additionally, Rhino’s geometry extraction feature is used to extract key geologic structural features, such as fault lines and lithology.

To calculate the spatial distance between the landslide site and the fault, and to assess the fault’s impact on the landslide, a geospatial computation method is applied. This method accounts for the surface curvature and calculates the true geodetic distance. This spatial distance is then used as a critical input for the “fault influence” factor in subsequent analyses.

**Figure 11:**
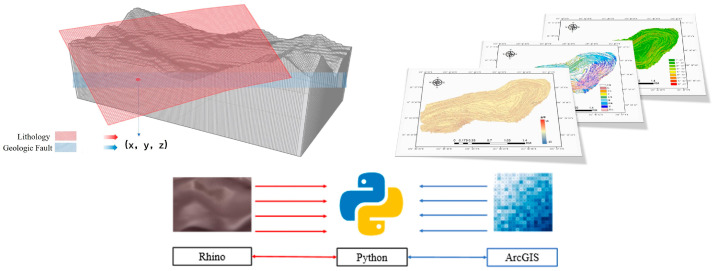
Information exchange between Rhino and ArcGIS.

For data integration, lithologic classification data are converted into a digital format suitable for analysis. The fault influence, lithologic features, and other topographic factors (such as slope, aspect, and curvature) are then fused using raster computation tools in the GIS, generating a new multifactor composite raster dataset. These newly generated raster data not only improve the completeness and accuracy of spatial representation but also provide more comprehensive data support for the analysis of geologic processes. As shown in Figure 11, this rich dataset provides high-quality, well-structured sample data for subsequent machine learning modeling.

## 6. Data-Driven Intelligent Landslide Prediction

### 6.1. Machine Learning Models and Evaluation Metric Introduction

To effectively predict the landslide risk on steep slopes, this paper selects five commonly used high-performance supervised learning models: random forest, logistic regression, K-nearest neighbors, support vector machine, and XGBoost.

Random forest is an ensemble learning method that builds multiple decision trees and combines their predictions to improve the accuracy and robustness of the model. It is well suited for handling nonlinear relationships and high-dimensional data. Logistic regression is a classical linear model commonly used for binary classification problems, predicting the probability of an event by fitting the logarithmic value of the data. The KNN algorithm is an instance-based learning method that predicts classification results by calculating the distance between samples. This algorithm is simple to understand but computationally expensive. SVM is a powerful classification algorithm that finds the optimal hyperplane in a high-dimensional space, making it suitable for nonlinear data. XGBoost is a slope boosting algorithm that has achieved excellent results in various fields due to its fast computational speed and strong performance, particularly in large-scale data processing and nonlinear modeling.

To evaluate the performance of each model in landslide prediction, common classification model evaluation metrics are used. The confusion matrix displays the relationship between the predicted results of a classification model and the actual labels, helping to calculate key metrics such as accuracy, precision, and recall to better understand the model’s misclassification patterns. The receiver operating characteristic (ROC) curve illustrates the model’s performance under different thresholds, while the area under the curve (AUC) value represents the model’s ability to distinguish between different categories. A higher AUC, closer to 1, indicates better model performance. The precision–recall curve shows the relationship between the precision and recall, making it particularly useful for handling unbalanced datasets. Balancing precision and recall is crucial in evaluating the model’s performance in a given task. These evaluation metrics enable a comprehensive assessment of different machine learning models in landslide prediction and provide a solid foundation for model selection.

### 6.2. Description of the Machine Learning Dataset

**Figure 12:**
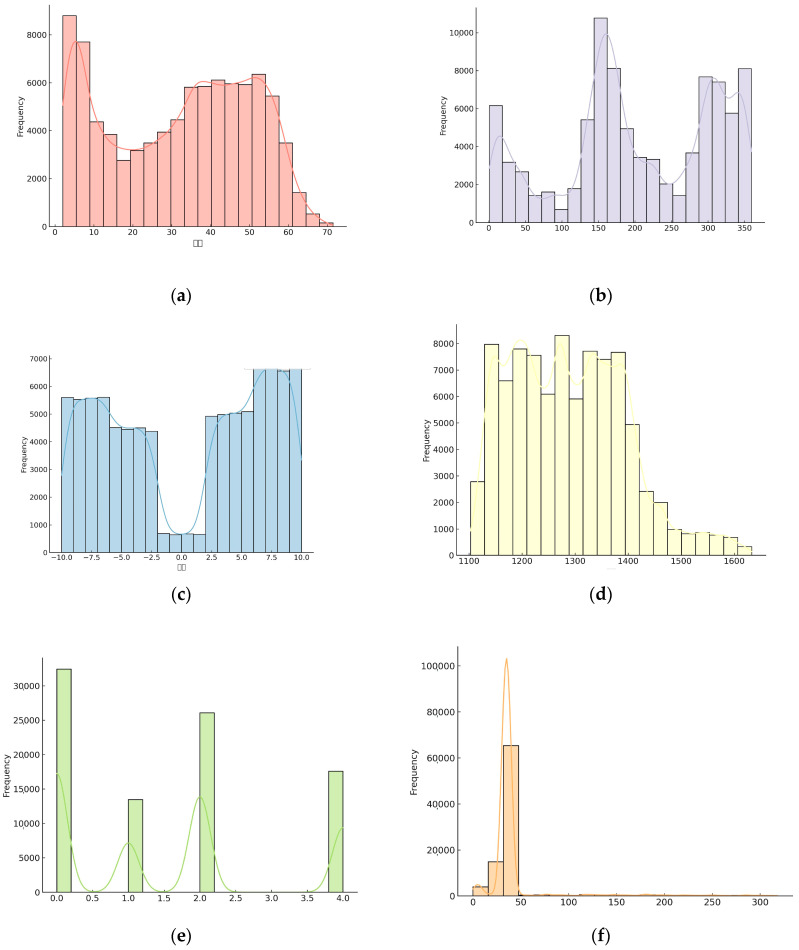
Description of the machine learning dataset: (**a**) slope; (**b**) aspect; (**c**) curvature; (**d**) elevation; (**e**) lithology; (**f**) fault spatial distance.

Merging the data resulted in the creation of a dataset containing five influencing factors: the slope, profile, slope, spatial distance, and lithology. However, categorical variables (e.g., lithology), which are crucial in representing geologic types, are typically non-numeric and cannot be directly input into machine learning models. To address this challenge, this study employed the one-hot encoding technique to convert categorical variables into binary numerical features. This process assigns distinct values to differentiate between the various lithologies, allowing the model to analyze each lithology category independently. A detailed description of the dataset is provided in Figure 12.

### 6.3. Evaluation of Machine Learning Model Accuracy

**Figure 13:**
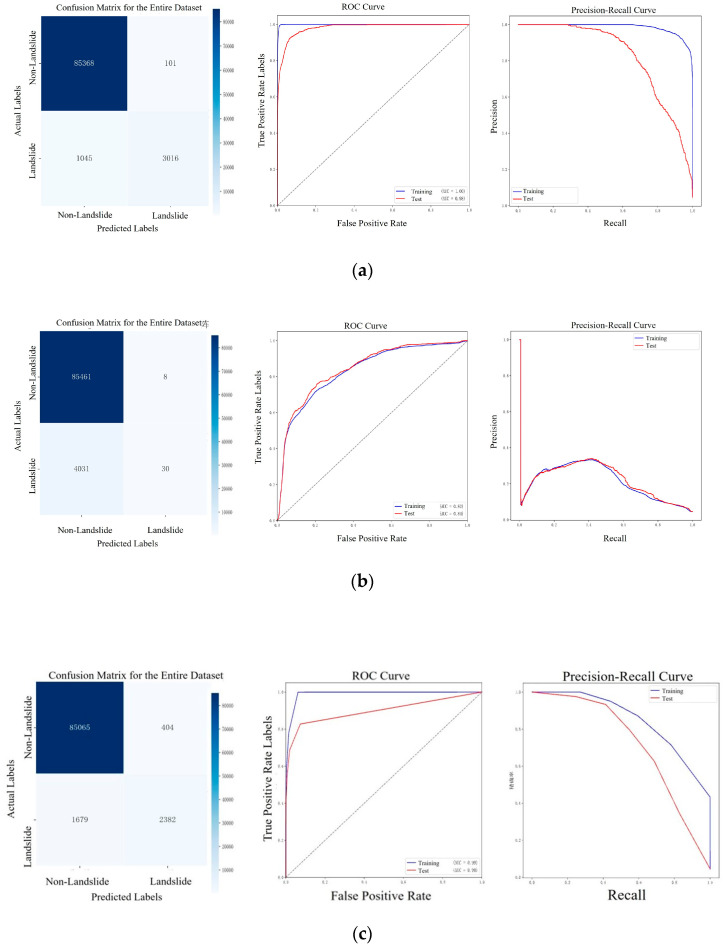

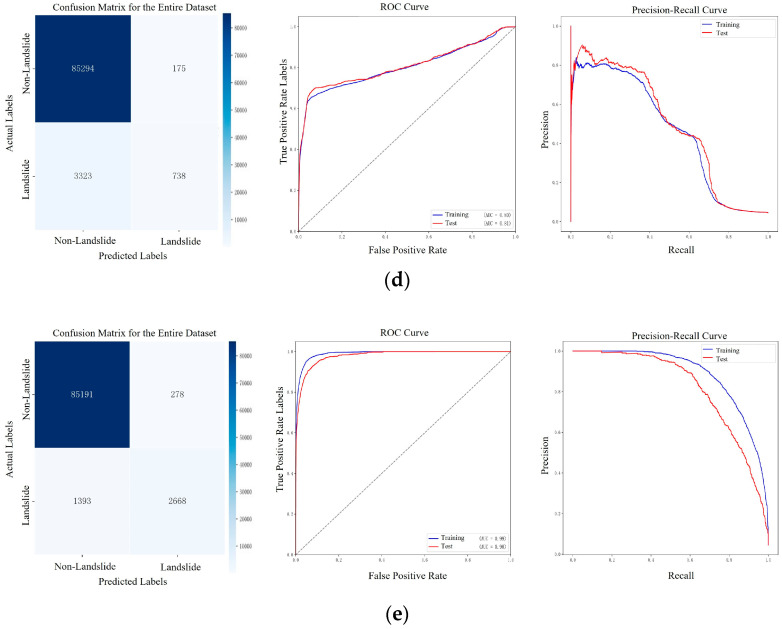
Machine learning results: (**a**) random forest; (**b**) logistic regression; (**c**) K-nearest neighbors; (**d**) support vector machine; (**e**) XGBoost.

In the landslide prediction task, the dataset was divided using an 80:20 split for training and testing. Five machine learning models were evaluated: random forest, logistic regression, K-nearest neighbors (KNN), support vector machine (SVM), and XGBoost. Their classification performance was assessed using four key metrics: the AUC, precision, recall, and overall balance. Detailed results are presented in Figure 13.

As shown in Figure 13, random forest and XGBoost demonstrated the best performance, with the AUC, precision, and recall values approaching 1. These models achieved high accuracy and a strong classification balance between landslide and non-landslide areas. Their ability to handle complex terrain and multidimensional feature data makes them particularly effective for this task.

Logistic regression also performed well in terms of the AUC and accuracy, but its recall value was relatively low. This indicates a higher likelihood of missing actual landslide occurrences, which limits its suitability for scenarios where detecting all potential hazards is critical. However, its simplicity and interpretability make it a viable choice in applications where false positives must be minimized. KNN showed the weakest performance across all metrics. Its limitations are more pronounced in large or complex datasets, and it is better suited for small-scale datasets with a uniform feature distribution. SVM delivered good results in terms of the AUC and precision but fell slightly short in its recall and overall classification balance. While effective in handling high-dimensional data, SVM is more appropriate for tasks with moderate classification complexity.

In conclusion, landslide prediction requires models that balance false negatives with the overall classification performance. Based on the comparative analysis, random forest and XGBoost offer clear advantages and are recommended as the preferred models for this application.

### 6.4. Landslide Risk Prediction and Spatial Distribution Characteristics Based on Soft Voting Strategy

Based on the analysis of the machine learning models, random forest and XGBoost perform significantly better than other models in landslide prediction. However, relying on a single model may not fully eliminate underfitting risks and may limit generalization, especially in complex prediction scenarios. To address these issues, this study proposes an ensemble approach using soft voting to combine random forest and XGBoost, aiming to further enhance the prediction performance and model stability.

Soft voting is a widely used ensemble learning strategy that combines the prediction probabilities of multiple classifiers to produce more reliable classification results. Each base model generates a probability distribution across categories, and the final prediction is produced by computing a weighted average of these probabilities. Unlike hard voting, which depends only on the majority decision of each model, soft voting leverages more detailed prediction information, improving both the accuracy and generalization.

**Figure 14:**
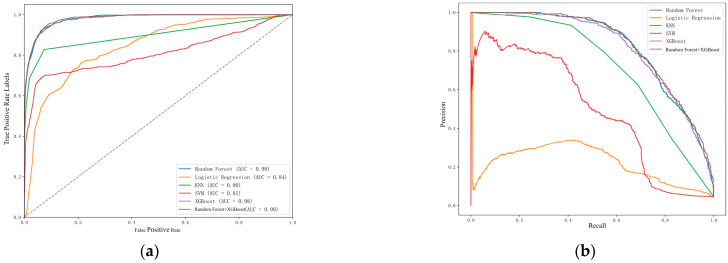
Model accuracy comparison: (**a**) ROC curve; (**b**) precision–recall curve.

As in Figure 14a,b, After integrating random forest and XGBoost through soft voting, the resulting coupled model shows significant advantages. It achieves a near-optimal ROC curve with an AUC value of 0.98, indicating strong discriminative abilities. The model is also robust to noise and can effectively manage high-dimensional data, redundant features, and interference. Even in imbalanced datasets, it maintains high precision and recall, demonstrating good adaptability to minority classes. Moreover, the model captures complex nonlinear relationships, addressing the limitations of traditional linear models. Its high parameter flexibility also allows for fine-tuning to adapt to various data characteristics and task requirements.

In this section, the use of soft voting to couple random forest and XGBoost provides an effective solution for landslide prediction. Random forest is particularly strong in handling nonlinear features and high-dimensional data, while XGBoost excels in slope boosting and capturing complex terrain-related features. Together, they complement each other and improve the overall performance.

**Figure 15:**
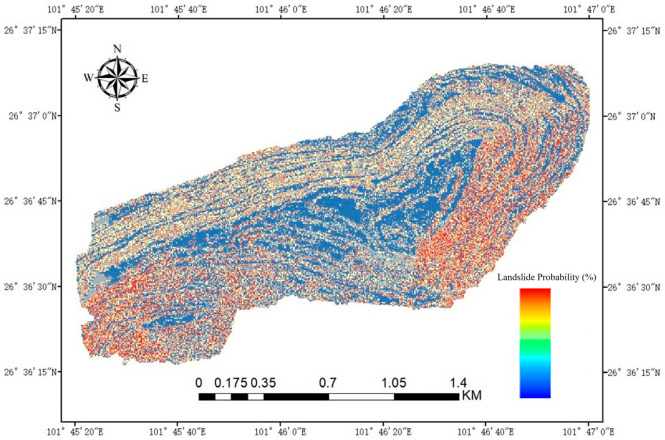
Landslide risk prediction for the entire mining area.

Based on the predictions from the coupled model, a total of 89,530 data points were generated, each containing a landslide probability value and corresponding XYZ spatial coordinates. These results not only provide specific probability values but also support comprehensive landslide risk assessment for the entire mining area. A landslide risk probability map can be created to visualize the spatial distribution of risk, as shown in Figure 15. Furthermore, if detailed classification criteria are available, the prediction results can be reclassified in ArcGIS to further refine the risk zones, enabling more accurate and targeted landslide risk assessment.

## 7. Numerical Simulation Analysis of Slope Stability in Key Areas Based on FLAC3D

### 7.1. Construction of the Numerical Simulation Model and Parameter Assignment

The model developed in Section 3, including the geological and fault information of the mine area, was imported into Rhino. Based on machine learning predictions, this section focuses on the southeastern part of the mine. The Griddle plugin in FLAC3D offers advantages in geometric restoration and mesh generation, using hexahedral elements for enhanced stability and accuracy in 3D slope simulations. It enables precise model partitioning and supports the assignment of material properties and grouping information, optimizing FLAC3D’s model management and settings. For the initial stress state, the self-weight loading method is used, where element densities are assigned to automatically generate the initial geostatic stress field.

To obtain the necessary parameters for FLAC3D simulation, this study was conducted in conjunction with the preliminary geological survey and field investigation from earlier stages. The primary research objects for the rock mechanics tests were iron ore, marble, coarse-grained gabbro, magnesium-grained gabbro, and quarry fine-grained gabbro. A total of 357 standard rock samples, each measuring 50 mm × 50 mm, were prepared for the rock mechanics tests. These included 85 iron ore samples, 145 marble samples, 69 coarse-grained gabbro samples, 18 medium-grained gabbro samples, and 40 fine-grained gabbro samples. These rock samples were then tested to determine the mechanical properties required for FLAC3D simulations.

**Figure 16:**
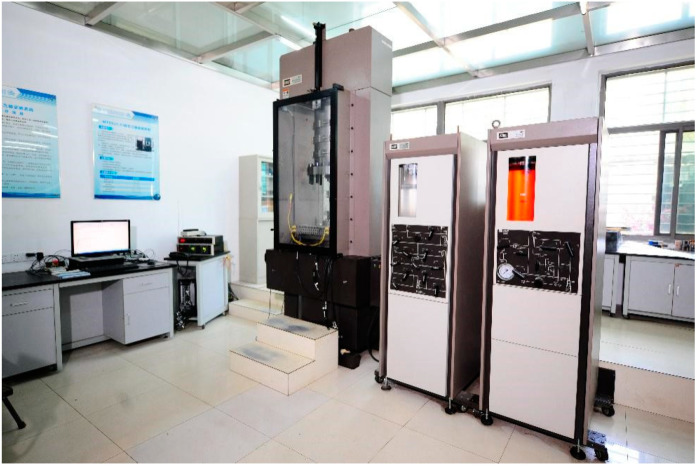
MTS815.03 rock mechanics testing system.

Triaxial compression tests were conducted using the MTS815.03 test system (Figure 16) to determine the modulus of elasticity, Poisson’s ratio, cohesion, and angle of internal friction of the rocks under different peripheral pressure levels. The specimens included iron ore, marble, coarse-grained gabbro, medium-grained gabbro, and fine-grained gabbro, with four specimens for each rock type, and the peripheral pressure was set at 5 MPa, 10 MPa, 20 MPa, and 40 MPa. The specimen size was 50 mm × 50 mm. Before the experiment, the specimens were wrapped with heat-shrinkable rubber sleeves to prevent hydraulic oil penetration, and pads were placed at both ends to reduce stress concentration. Loading was controlled by axial displacement at a rate of 0.005 mm/s. After stabilizing the peripheral pressure, axial stress was applied until failure, and data for the entire process were recorded.

Based on the results, the triaxial compressive strength was calculated, and the axial stress–axial strain and axial stress–radial strain curves were plotted. The modulus of elasticity and Poisson’s ratio were determined in the elastic phase by selecting the linear portions of the curves. The elastic modulus was calculated as the slope of the stress–axial strain curve, and Poisson’s ratio was derived from the ratio of axial strain to radial strain.

Additionally, using the Mohr–Coulomb damage criterion, the peak stresses under different peripheral pressure levels were fitted to a linear relationship, and the cohesion and internal friction angle were obtained through regression analysis. This evaluation provided key mechanical properties of the rock, as shown in Table 1.

### 7.2. Numerical Simulation Analysis Based on FLAC3D

In the excavation simulation, material removal was performed step by step, unloading corresponding units in sequence to simulate stress release and slope deformation trends. The exact excavation locations for each stage were determined based on the mine’s work plan and the shape of the already excavated benches. The X, Y, and Z coordinates in the FLAC3D model represent the coordinates within the model, indicating positions in the simulation space. The model coordinates are in meters (m), consistent with actual engineering dimensions, ensuring the model’s physical accuracy and engineering relevance.

The simulation of the excavation process reflects the real working conditions of the open-pit quarry. The initial step (T0 stage) involved calculating the initial geostatic balance, with no material removed, establishing a stable starting condition. Following this, a staged excavation approach was adopted. In the T1 stage, excavation was performed within the ranges X: 0–180, Y: −930 to −1223, and Z: 598–588. In T2, the excavation extended the Z range to 588–578. T3 continued excavation to Z: 578–568, maintaining the same X and Y ranges. T4 progressed the excavation to Z: 568–558, with further deepening. In T5, a new excavation zone was defined within X: 0–140, Y: −986 to −1223, and Z: 558–528. In T6, excavation narrowed the area to X: 0–100, Y: −1086 to −1223, and Z: 528–478. Finally, in T7, excavation focused on the deepest and smallest range, X: 0–60, Y: −1186 to −1223, and Z: 478–418. This progressive excavation strategy started from broader, shallow zones and gradually extended to greater depths, allowing a detailed analysis of how each excavation stage affected the overall slope stability and stress redistribution in the rock mass. This approach provides valuable insights into deformation patterns, potential failure mechanisms, and areas of concern throughout the excavation process.

In FLAC3D, the FOS is calculated using the limit equilibrium method to assess the slope instability risk. The maximum deformation displacement is calculated based on the material’s mechanical properties, reflecting the extent of rock mass deformation under external loads.

**Figure 17:**
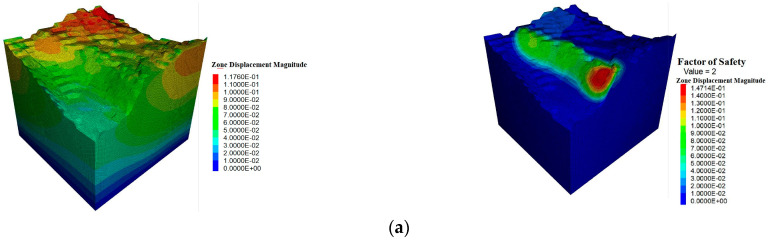

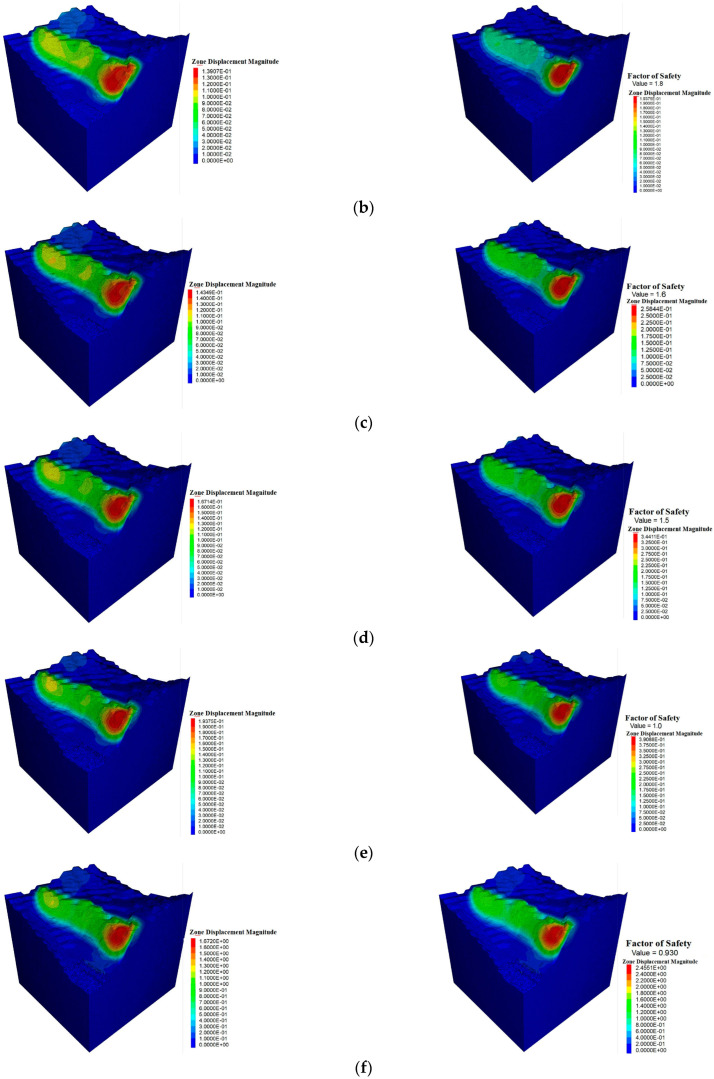

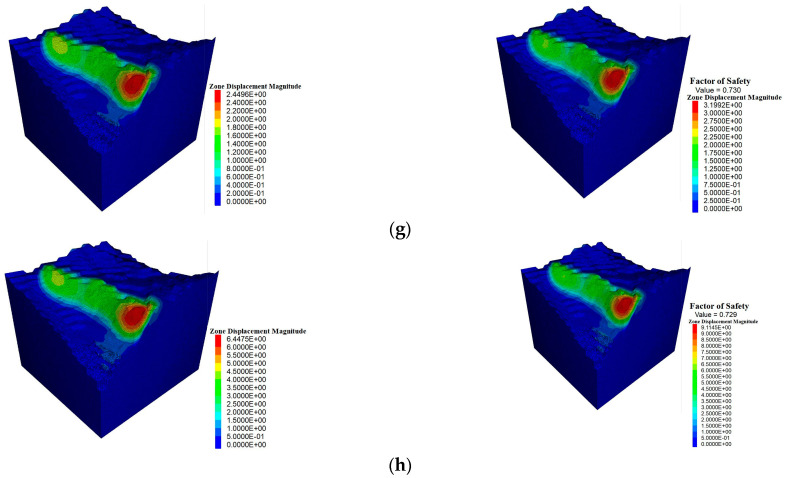
Displacement contour maps at different stages and slope safety factors. (**a**) T0; (**b**) T1; (**c**) T2; (**d**) T3; (**e**) T4; (**f**) T5; (**g**) T6; (**h**) T7.

As shown in Figure 17, In the T0 stage, the model is stable with no excavation, showing maximum displacement of 0.12 m, mostly concentrated in the shallow surface layer. The factor of safety (FOS) is 2.0, indicating stability with minimal deformation and no plastic zone development. In the T1 stage, the displacement increases slightly to 0.13 m, and the FOS drops to 1.8, signaling a slight decrease in stability. By T2, the displacement reaches 0.14 m, with a local plastic zone forming and the FOS dropping to 1.6, indicating some reduction in stability but still within acceptable limits. In T3, the displacement increases to 0.17 m, and the FOS further decreases to 1.5, showing signs of tensile damage. During T4, the maximum displacement reaches 0.19 m, and the FOS drops to 1.0, approaching critical instability. The displacement concentration increases, with the damage mode shifting to shear, signaling increased risk. In T5, the displacement rises to 0.24 m, and the FOS drops to 0.93, indicating instability. The plastic zone expands, and shear slip occurs. By T6, the displacement dramatically increases to 0.91 m, with the FOS at 0.73, signaling serious instability and a high risk of landslides. In T7, the displacement reaches 6.45 m, confirming total destabilization, with the FOS remaining at 0.73. The plastic zone extends throughout the excavation area, and collapse is imminent without support or backfilling.

**Figure 18:**
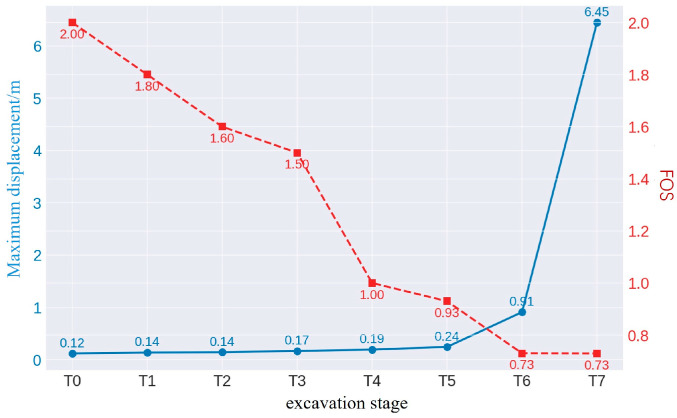
Trends of displacement and slope safety factor variations.

As shown in Figure 18 trends of the maximum displacement and FOS across the excavation stages. As the excavation deepens, the displacement increases, especially between T6 and T7, where it rises sharply to 6.45 m. The FOS consistently decreases, dropping below 1.0 after T5, indicating a substantial reduction in slope stability and an increased risk of collapse.

**Figure 19:**
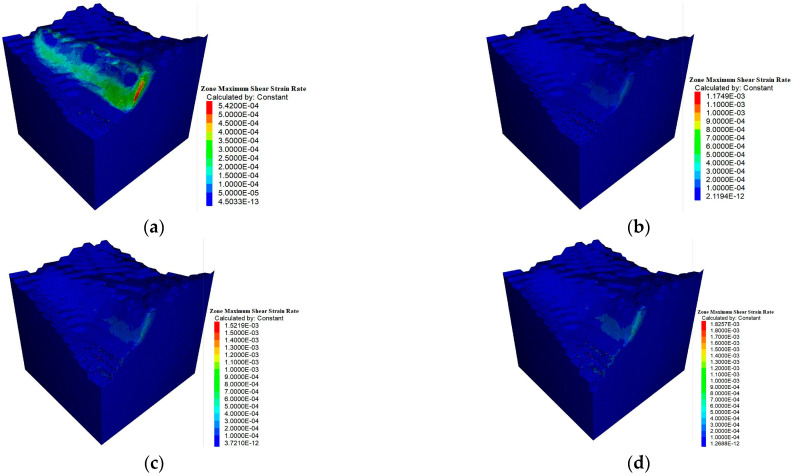
Shear rate change. (**a**) T4; (**b**) T5; (**c**) T6; (**d**) T7.

The shear rate is obtained by calculating the rate of change in shear strain for each element. Through the update of the stress and strain in the numerical simulation, FLAC3D can compute the deformation rate of the material under shear stress, predicting the occurrence of landslides. As shown in Figure 19a, In the T4 stage, the shear rate jumps to 5.42 × 10^−4^ s^−1^, with the shear band expanding significantly. This indicates the rapid development of the plastic zone, suggesting that the rock mass is nearing its ultimate strength and its stability is beginning to decline, marking a critical point for potential failure.

As shown in Figure 19b, In the T5 stage, the shear rate increases further to 1.17 × 10^−3^ s^−1^, almost double that of T4, signaling the onset of accelerated deformation. The shear band continues to expand, and more high-shear areas appear, indicating that the rock mass may have entered the slip stage, with the instability risks rising sharply.

As shown in Figure 19c, In T6, the shear rate peaks at 1.52 × 10^−3^ s^−1^, and the shear band forms a continuous structure. This suggests that local sliding has occurred and continuous shear failure is in progress. The failure mode shifts from localized deformation to large-scale sliding, greatly increasing the overall risk of instability.

As shown in Figure 19d, In the T7 stage, the shear rate reaches its highest value of 1.83 × 10^−3^ s^−1^, indicating that the rock mass is fully unstable. The shear failure band is now pervasive, signaling that the failure has reached its maximum extent.

The plastic zone is where the rock undergoes irreversible deformation once the stress exceeds its yield strength. Below the yield limit, the rock deforms elastically and can return to its original shape. However, if the stress surpasses the yield strength, plastic deformation occurs, potentially forming a shear zone or rupture surface. In open-pit mine stability analysis, studying the plastic zone helps to identify failure-prone areas and detect early signs of instability, aiding in slope management and safety measures.

From T0 to T3, there is little to no plastic deformation in the rock mass. This is because the stress levels are still low and have not exceeded the yield strength of the rock. In T0, the rock is stable, and the deformation is elastic. As stress builds up from T1 to T3, there are small areas of local stress concentration, but, overall, the rock mass remains elastic, and no significant plastic zones form. The shear rate remains low, which means that the deformation is minimal, and the rock is still stable, as indicated by the high factor of safety (FOS). Therefore, during these stages, the rock mass mainly deforms elastically and has not entered the plastic damage stage.

**Figure 20:**
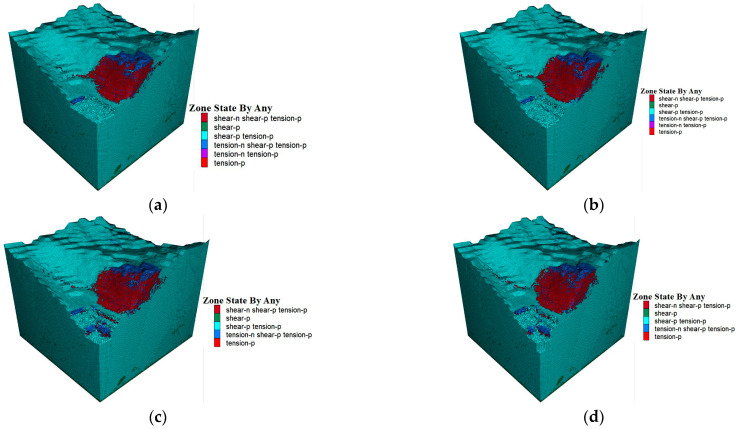
Distribution of plastic zone location. (**a**) T4; (**b**) T5; (**c**) T6; (**d**) T7.

Starting from the T4 stage, the plastic zone begins to appear and expands over time (Figure 20). In the T4 stage, red and blue areas emerge in local regions, indicating shear and tension damage, especially at the foot of the slope and the slope face. The rock’s bearing capacity starts to decrease, and the plastic deformation area grows.

In T5, the plastic zone grows significantly, with the red area expanding further, showing that the shear damage has worsened and the rock mass’s structural integrity is being affected. By T6, the plastic zone continues to develop, with both the shear and tensile damage areas increasing. The plastic zone starts to penetrate, indicating that an internal shear zone is forming and the slope may be nearing critical instability. In T7, the plastic zone fully penetrates, and the red area greatly increases, meaning that the rock mass has entered full plastic damage, and the slope is now at serious risk of landsliding. In short, the appearance and growth of the plastic zone reflect the rock mass’s stability evolution. From T4 onward, the plastic zone expands until T7, showing that the slope transitions from stable to destabilized. This analysis is crucial in assessing the safety of mine slopes.

Based on the combined simulation results regarding the FOS, shear rate, and plastic zone, it is highly likely that a landslide disaster will occur in the southeast slope of the T4 stage. Considering the actual operational conditions, it is recommended to implement support measures at the location where the plastic zone develops before the T4 stage to prevent a landslide. Additionally, enhanced monitoring should be conducted in key areas.

## 8. Conclusions

This paper establishes a fast and accurate landslide prediction method for open-pit mine slopes, considering the risks associated with data acquisition for high and steep slopes. An innovative approach is presented by applying UAV-based terrain-following flight technology for data collection, while integrating multi-source data to establish a refined 3D geological model. This approach addresses two key challenges in applying GISs to open-pit mines, improving the mine’s landslide prediction monitoring and forecasting capabilities. By establishing a dataset through 3D spatial coordinate correspondence, data exchange between the 3D geological model and GIS is achieved. Subsequently, machine learning methods are introduced for intelligent landslide prediction across the entire mine area. Numerical simulations are then employed to simulate excavation in key areas, predicting the timing and location of landslides during actual mining operations. This study not only highlights the great potential of modern technology in ensuring safety in open-pit mining but also provides innovative ideas and methods for future research in related fields. The specific conclusions are as follows.

(1) This study highlights the application of UAV-based terrain-following flight technology on high and steep slopes in open-pit mines. By combining fieldwork and indoor processing, high-quality 3D models were successfully obtained. This technology provides an effective solution for real-time landslide monitoring, particularly in hazard area identification and early warning, offering higher accuracy compared to traditional methods.
(2) This research successfully applied a GIS to landslide analysis in open-pit mines. By utilizing the 3DMine and Rhino 8 software, UAV-acquired aerial data were integrated with geological information to create a refined 3D geological model, which included lithology and fault data.
(3) A GIS was used to analyze relevant influencing factors. These factors were linked to the lithology and fault effects in the 3D geological model through spatial coordinate correspondence, creating a machine learning dataset. Multi-source data fusion provided an accurate sample dataset for subsequent machine learning, significantly improving the accuracy of landslide prediction. Compared to traditional single-source methods, this approach better reflects the multidimensional characteristics of the mining environment.
(4) Several machine learning algorithms were applied, with random forest and XGBoost demonstrating strong data processing and prediction capabilities. To enhance the stability and accuracy of the models, a soft voting method was used to integrate random forest and XGBoost. This integration makes the model particularly suitable for handling complex terrain and high-dimensional data. Based on the prediction results from the machine learning models, a landslide probability distribution map for the entire mining area was generated, providing an overall landslide prediction for the mine.
(5) Based on the machine learning results, the southeastern part was selected as the focus area for numerical simulation. Simulating the actual excavation process, the model predicted a sharp change in the FOS starting at the T4 stage, recording the maximum displacement, the shear rate, and the location of plastic zone formation at each stage. The numerical simulation method provided a clear prediction of the location and timing of landslide formation in the key area.

## Figures and Tables

**Table 1:** FLAC3D parameter values.

Lithology	Unit Weight (kN/m^3^)	Cohesion (MPa)	Friction Angle (°)	Modulus of Elasticity (GPa)	Poisson’s Ratio
Ore	34	48.2	40	82.49	0.20
Marble	32	41.8	38.8	48.56	0.21
Coarse-Grained Gabbro	31	31.7	46.3	52.04	0.26
Medium-Grained Gabbro	31	25.9	53.3	75.32	0.17
Fine-Grained Gabbro	31	37.8	47.3	52.04	0.26
Faults	25	200	26	0.09	0.3

## Data Availability

The raw data supporting the conclusions of this article will be made available by the authors on request.
